# Integrated structural analysis for geothermal exploration: A new protocol combining remote sensing and aeromagnetic geophysical data

**DOI:** 10.1016/j.mex.2025.103189

**Published:** 2025-01-28

**Authors:** Jawad Rafiq, Israa S. Abu-Mahfouz, Konstantinos Chavanidis, Pantelis Soupios

**Affiliations:** Department of Geosciences, King Fahd University of Petroleum and Minerals (KFUPM), Saudi Arabia

**Keywords:** Structural geology, Sustainability, Geothermal energy, Integrated Surface-Subsurface Structural Analysis for Geothermal Exploration

## Abstract

Geothermal energy holds significant potential as a sustainable and clean source, yet efficient exploration methodologies remain critical for identifying viable sites. This paper presents a novel protocol for the identification and analysis of structural lineaments in geothermal fields, crucial for coherent geothermal exploration. The approach integrates surface data from remote sensing and data from airborne magnetic geophysical surveys that provide information on the subsurface structures, to analyze structural lineament density analysis, orientation, and high permeable zones, and assess geothermal potential. By combining information from these two sources, the study demonstrates the relationships between structural lineaments and areas of high permeability, shedding light on geothermal resource distribution. This twofold structural analysis not only enhances our ability to identify potential geothermal sites but also contributes to a deeper understanding of the geological factors influencing geothermal reservoirs. This integrated approach advances geothermal exploration in line with the global shift towards sustainable energy.

• The straightforward nature of the approach enables its versatile application for predicting various geological processes beyond geothermal exploration.
• The application of this protocol is accessible to a broader audience of researchers, as it does not require knowledge of programming language.
• The results obtained from this approach demonstrate high predictive performance, underscoring reliability in identifying and analyzing structural elements in geothermal fields.

## Graphical abstract

**Figure:**
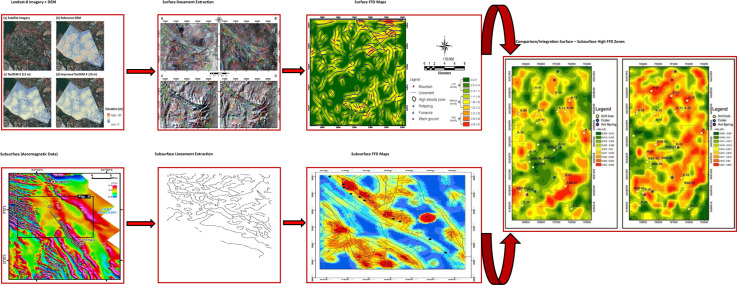

Specifications table

**Table:** 

Subject area:	Energy
More specific subject area:	Structural Geology for Geothermal Exploration
Name of your protocol:	Integrated Surface-Subsurface Structural Analysis for Geothermal Exploration
Reagents/tools:	• Landsat-8 imagery and Digital Elevation Models (DEMs) of 30 m and 12 m. • Catalyst version of PCI Geomatics software: (https://catalyst.earth/about/) • Seequent's Oasis montaj software (https://www.seequent.com/) and CET (Centre for Exploration Targeting) Grid Analysis extension.
Experimental design:	Integration of remotely sensed and aeromagnetic geophysical data for structural analysis (surface and subsurface) of a geothermal reservoir.
Trial registration:	For surface lineament analysis: (https://catalyst.earth/about/) For subsurface lineament analysis: (https://www.seequent.com/)
Ethics:	Not applicable.
Value of the Protocol:	• The straightforward nature of the approach enables its versatile application for predicting various geological features/processes beyond geothermal exploration. • The application of this protocol is accessible to a broader audience of researchers, as it does not require knowledge of programming language. • The results from this methodology demonstrate high predictive performance, underscoring reliability in identifying and analyzing structural elements in geothermal fields.

## Description of protocol

### Surface lineament analysis from remote sensing data

Landsat-8 imagery and Digital Elevation Models (DEMs) of 30 m and 12 m respectively served as primary datasets for surface lineament extraction. Landsat-8 imagery provides high-resolution multispectral data, while DEMs offer crucial elevation information. The lineament extraction process was conducted using the Catalyst version of PCI Geomatics software (https://catalyst.earth/about/), known for its advanced capabilities in remote sensing and geospatial analysis. Directional filters were applied to enhance lineament detection, utilizing a three-by-three convolution filter window to obtain the shortest lineament length. Automated extraction from DEMs and Landsat-8 imagery was performed to create a comprehensive lineament map. The Line Module settings were optimized for discontinuity extraction, involving thresholding, edge detection, and extraction of curves (Table 1). The retrieved lineaments were used to generate a synthetic lineament map.

**Table 1:** Main parameters used in the remote sensing software (PCI Geomatics) for automated (digital) lineament extraction.

Parameters	Values
RADI	10
DTHR	20
FTHR	3
GTHR	100
ATHR	30
LTHR	30

To detect minor lineaments, those repeated more than one time were systematically removed, preventing redundancy in the synthetic map. To do so geographic information system (GIS) techniques were employed to eliminate lineaments resulting from hydrographic networks, human activities, and illogical features. Larger lineaments not captured by the automated process were manually marked on the DEM. Finally, lineament density maps were constructed, providing a spatial representation of lineament distribution across the study area.

The Fault Fracture Density (FFD) method proposed by Taucare et al. [1] and utilized by Nahli et al. [2], Arrofi et al. [3], Arrofi and Abu-Mahfouz [4], and Rafiq et al. [5] was used to define distinct structural lineament density zones within the DEM. To calculate fracture density within the area of study, the FFD method involved dividing the length of identified lineaments (L, in km) by the area of the corresponding cell (A, in km^2^). The mathematical expression for fracture density (FD) is given by Eq. (1):

(1) $$\mathrm{FFD}=\frac{Length\;of\;Lineaments\;\left(km\right)}{Area\;of\;the\;Cell\;(km^{2})}$$

### Subsurface lineament analysis from aeromagnetic geophysical data

A commonly applied methodology for structural analysis of a study area involves the utilization of magnetic geophysical data. In this study, the Centre for Exploration Targeting (CET) Grid Analysis, an extension of Seequent's Oasis montaj software (https://www.seequent.com/), was used for identifying subsurface structural lineaments from the analysis of airborne magnetic data. The CET Grid Analysis module allows for the automatic detection of lineaments in the gridded potential field (magnetic and gravity) data, minimizing the time required for grid interpretation. The suite of CET algorithms was developed by the University of Western Australia [6] and provides useful tools for texture enhancement, phase analysis, structural complexity analysis, and edge and threshold detection by estimation of the lineament trends [[7], [8], [9]]. The extension is widely used in the first stages of natural resource exploration projects where data from potential field geophysical methods are usually acquired, especially in mineral exploration cases (e.g., [[10], [11], [12]]). It allows the identification of sites and zones where resources might accumulate (e.g., the crossing points of dikes where precious minerals are encountered) indicating potential structures for detailed future studies by applying drilling or other geophysical techniques.

Initially, the Texture Analysis part of the CET algorithm calculates the standard deviation, providing an estimate of the variations in the data, as shown mathematically in Eq. (2):

(2) $$\mathrm{Standard}\;\mathrm{deviation}=\sqrt{\frac{\Sigma {\left(X-\bar{x}\right)}^{2}}{n-1}}$$

where *n* is the total number of observations, *X* is the data distribution value and $\bar{x}$ is the sample mean value.

Highly contrasting values, as compared to the background signal, indicate the possible presence of significant structural characteristics [6].

The Lineation Detection algorithm was then applied, using the methods developed by Kovesi [13] as the default on the software. According to the nature of the study, the Phase Symmetry or the Phase Congruency routine can be selected. The two-dimensional gridded data are split into one-dimensional profiles; these profiles are analyzed over multiple directions and scales so that strong symmetrical responses are produced in every direction except those parallel to the linear structures, thus enhancing their appearance and main trends. This procedure allows for the mapping of linear features that include lithological boundaries and structural features, such as faults and dikes.

SymmetryPoint=MinimumorMaximumValueintheFrequencyDomain

Afterward, the Amplitude Thresholding routine is applied as part of the Lineation Vectorization algorithm, which identifies ridges from the processed data and removes the noise. The value of the threshold should be set according to the detail of the study. The use of higher values indicates the locations of the major regional lineaments, while lower values will also detect lineaments that correspond to local features. The result is a map that shows thinned straight-linear features that can be further used for mining or structural studies.

After the vectorization of lineaments, the next phase of the analysis includes the computation of fault and fracture density (FFD) maps. This process entails quantifying the number of fault and fracture occurrences within defined grid cells or spatial units using the Skeleton-to-Vector analysis results of CET. The density analysis is often expressed mathematically as shown in Eq. (3):

(3) $$\begin{array}{c} \mathrm{Density}=\frac{\mathrm{Numb}\mathrm{er}\;\mathrm{of}\;\mathrm{Line}\mathrm{amen}\mathrm{ts}\;\mathrm{in}\;a\;\mathrm{Cell}}{\mathrm{Area}\;\mathrm{of}\;\mathrm{the}\;\mathrm{Cell}} \end{array}$$

### Protocol summary (Step-by-step)

**Fig. 1:**
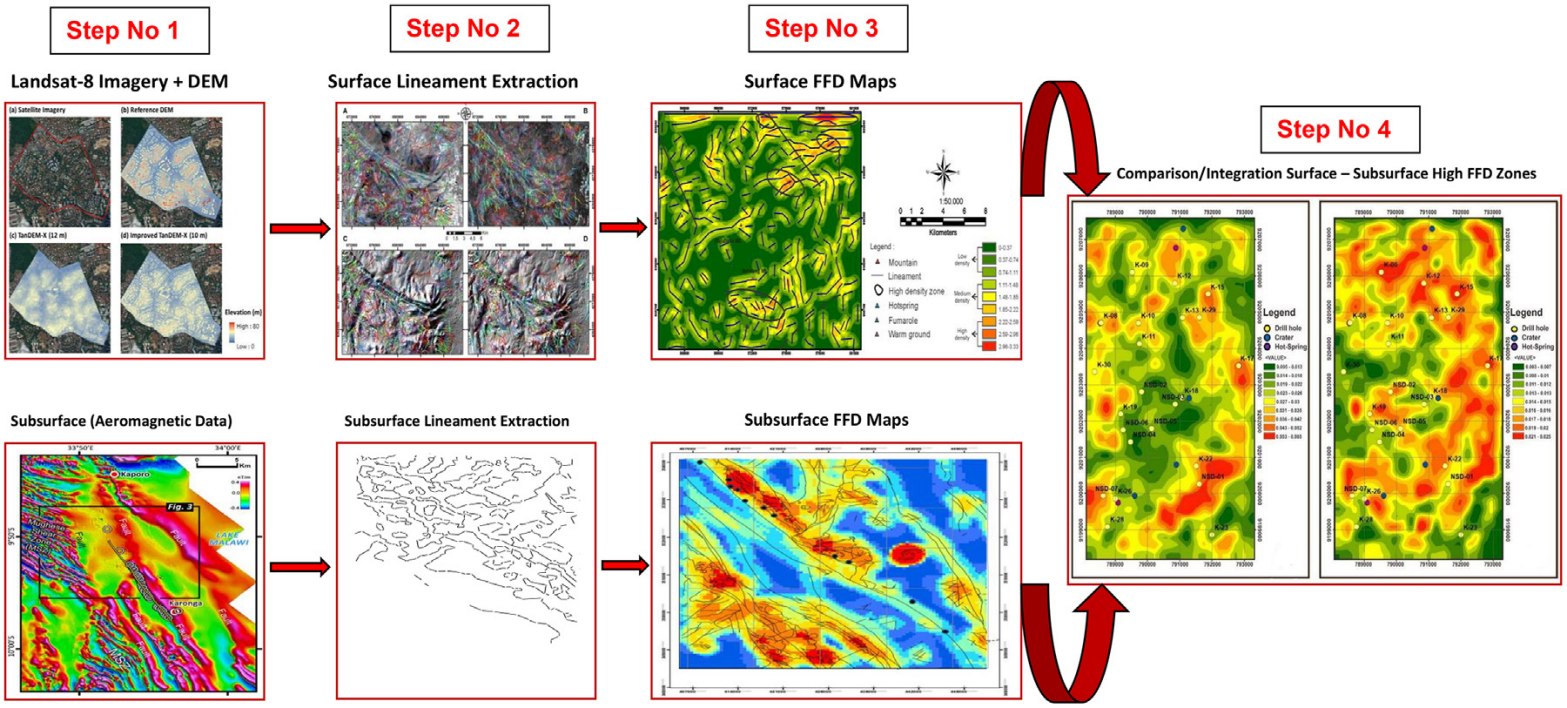
Workflow of the new protocol for surface-subsurface structural analysis of the geothermal field using integrated remotely sensed and aeromagnetic geophysical data. *Note:* the magnetic map and Landsat imagery used in this figure are for protocol demonstration purposes only.

1. **Data Acquisition and Preprocessing**
  ○ Begin by acquiring Landsat-8 imagery, Digital Elevation Models (DEMs), and airborne magnetic data.
  ○ Proceed with data preprocessing, which includes applying radiometric corrections, smoothing the DEMs, and standardizing the magnetic data.
2. **Lineament Extraction**
  ○ **Surface Lineaments:** Utilize Catalyst PCI Geomatics software to automate the extraction of surface lineaments using directional filters, complemented by manual extraction with ArcGIS software. Fine-tune the extraction parameters, remove redundant lineaments, and eliminate irrelevant features to ensure accuracy.
  ○ **Subsurface Lineaments:** Detect subsurface structural lineaments through CET grid analysis in Seequent Oasis Montaj. Enhance textures and apply edge detection techniques to identify both major and minor lineaments.
3. **Lineament Density Mapping**
  ○ Create Fault Fracture Density (FFD) maps for both surface and subsurface lineaments by calculating the lineament length per grid cell area using ArcGIS software.
  ○ Identify high-density zones, which indicate regions of increased permeability within the study area.
4. **Integration & Comparison**
  ○ Integrate the surface and subsurface lineament data, placing particular emphasis on the high FFD zones identified in the previous step.
  ○ Compare the trends of the lineament orientations to uncover correlations and discrepancies, culminating in the development of a comprehensive structural model (refer to Fig. 1).

## Rationale for methodology and tool selection

### Surface lineament analysis from remote sensing data

We chose Landsat-8 imagery and Digital Elevation Models (DEMs) due to their high spatial resolution and comprehensive coverage, which are essential for effective lineament extraction. Catalyst (the latest version of PCI Geomatics software) was selected for its advanced capabilities in processing satellite imagery and DEMs. Specifically, the use of directional filters in this software enhances the detection of lineaments by focusing on preferred orientations, significantly improving extraction outcomes compared to basic edge-detection algorithms. Additionally, the automated extraction process reduces human error and inefficiencies associated with manual mapping, while the GIS techniques allow for the systematic removal of non-relevant features, thus improving the accuracy of our synthesized lineament map.

### Subsurface lineament analysis from airborne magnetic geophysical data

The CET Grid Analysis was employed for subsurface lineament analysis primarily because it is purpose-built for extracting linear features that correspond to geo-tectonic elements in gridded potential field geophysical data. This tool automates the identification of lineaments, a crucial advantage over traditional manual interpretation methods, which are often time-consuming and are highly dependent on the interpreter's experience. The suite of CET algorithms incorporates texture enhancement and edge detection processes in discrete steps, which are pivotal for discerning subtle geological features that may indicate geologic contacts, faults, or fractures in the subsurface.

The initial standard deviation calculation performed within the CET analysis is particularly advantageous because it quantitatively assesses variations in magnetic data, allowing for the identification of significant structural features against background noise. Furthermore, the Lineation Detection and Amplitude Thresholding routines enable a multi-scaled analysis that can effectively differentiate between major and minor lineaments. This flexibility means that the analysis can be tailored to the specific geophysical characteristics of the study area, optimizing the detection process.

### Data integration & validation

Surface lineament extraction from Landsat-8 imagery and DEM can be compared with subsurface lineaments identified from aeromagnetic data. The integration aimed to identify potential correlations between surface and subsurface structural features indicative of geothermal reservoirs. Furthermore, fault fracture density (FFD) analysis enables us to distinguish the prevalence of a substantial quantity of structural elements (i.e., lineaments), such as faults and fractures, within the studied area.

The zones of high fault fracture density indicate the presence of areas with high permeability within the geothermal system. In turn, it allows us to pinpoint the exact locations for targeting geothermal resource exploitation.

Statistical validation of the results was conducted to evaluate the efficacy of our protocol in extracting structural lineaments and identifying high-permeability zones. This included utilizing numerical measures of predictive performance, including metrics such as precision.

### Case study (Wadi Al-Lith, western Saudi Arabia)

The protocol has been effectively applied to the Wadi Al-Lith region, a promising geothermal field in western Saudi Arabia. By combining remote sensing, airborne magnetic data, and advanced processing techniques, the study provides valuable insights into the surface and subsurface structural characteristics that influence geothermal potential. The findings from this approach can be directly utilized to guide further exploration and development of geothermal resources in Wadi Al-Lith and similar tectonically active regions globally.

### Study area description

Wadi Al-Lith, situated on a tectonic plate boundary along the Red Sea coast, is a highly promising geothermal exploration region within the Arabian Shield due to its tectonic activity and the presence of numerous hot springs, including Ain Al Harrah and Ain Markub [5]. The study area, covering approximately 1615.73 km², consists of diverse geological formations, including Precambrian basement rocks, felsic intrusions, and Quaternary sediments. The complex structural setting, marked by faults, dikes, and fractures, offers a unique opportunity to apply an integrated structural analysis using our new protocol that combines remote sensing and aeromagnetic geophysical data.

### Methodology

The study utilized remote sensing and aeromagnetic data to map surface and subsurface structural lineaments. Surface structural lineaments were extracted using high-resolution Landsat-8 imagery and digital elevation models (DEMs) at 12 m and 30 m resolutions. Advanced digital processing methods in PCI Geomatics and GIS-based manual interpretation were employed to generate lineament maps and density zones.

For subsurface structural lineaments, airborne magnetic data were utilized. The data are part of a wider dataset that covers the entire Arabian Shield and the Phanerozoic cover in western and central Saudi Arabia [[14], [15]] and it was provided in reduced-to-pole (RTP) format by the Saudi Geological Survey (SGS). The data were processed using Oasis montaj and its CET Grid Analysis extension to extract the linear features of the subsurface. Lineament orientation analysis was performed with rose diagrams to compare surface and subsurface structural trends.

## Results & discussions

**Fig. 2:**
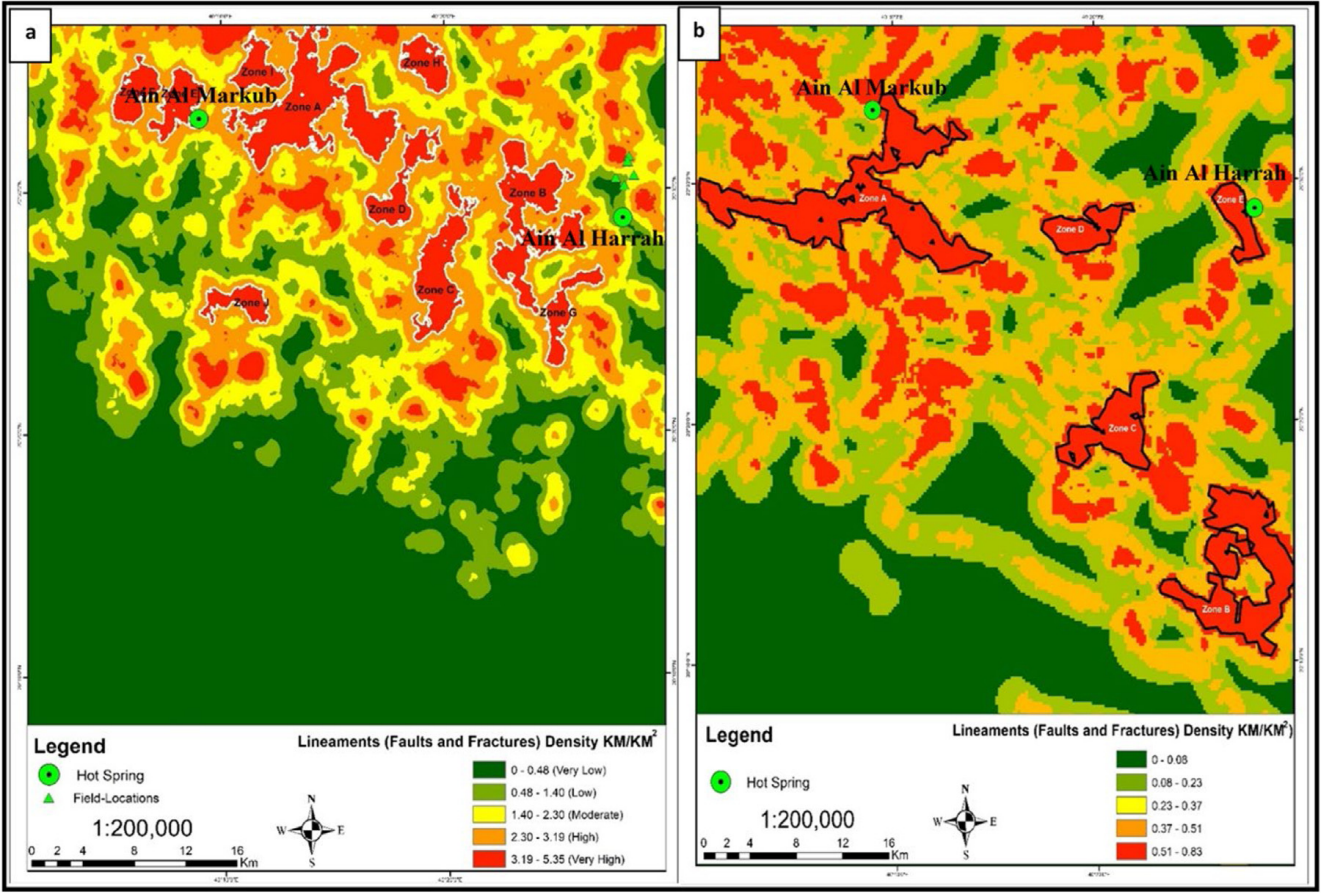
Surface and Subsurface lineament density maps of the study area (Al-Lith, Saudi Arabia): **a)** Density map of the surface lineaments extracted from the DEM. **b)** Density map of the subsurface lineaments extracted from the aeromagnetic data.

The integrated analysis of surface and subsurface lineaments revealed significant structural features indicative of geothermal potential in the Wadi Al-Lith region. A total of 1467 surface lineaments, spanning 72.55 km, were extracted, with orientations primarily following N-S, NE-SW, and NW-SE trends, which align with the regional tectonic regime. Ten high-density zones were identified, with Zone A exhibiting the highest density and closely correlating with thermal manifestations in the field. Subsurface analysis revealed five high-density zones close to the hot spring sites, predominantly oriented along NW-SE and NE-SW trends, with Zone A covering 88 km², showing a strong correlation with the highest surface density zones and proximity to the Ain Markub hot spring (Fig. 2). This suggests strong surface-subsurface connectivity and highlights the region's high permeability zones. The consistency between surface and subsurface structural trends emphasizes the interconnected fracture networks crucial for geothermal fluid circulation. Zones A and B, with high fault fracture density values and proximity to hot springs, are prime targets for further geothermal exploration.

The integration of remote sensing and aeromagnetic data in our study presents a significant advancement in structural analysis techniques applied to geothermal exploration. Previous studies, notably those by Nahli et al. [2] and Arrofi et al. [3], have adeptly employed the FFD methodology, proposed by Taucare et al. [1], to map lineament density in various geological contexts. Moreover, Chavanidis et al. [7] used airborne magnetic data to study the subsurface lineament distribution and density in the wider Wadi Al-Lith region, which provided useful insights for the geothermal exploration efforts in the area. However, our study enhances the FFD analysis through the integration of high-resolution Landsat-8 imagery, aeromagnetic geophysical data analysis, and advanced digital processing techniques to map surface and subsurface structural lineaments in geothermal fields. Notably, while Nahli et al. [2] focused primarily on identifying surface lineaments linked to hydrocarbon potential without the integration of subsurface data, our protocol extends this analysis by incorporating a comprehensive view of both surface and subsurface lineaments.

The outcomes of our protocol indicate higher fidelity in identifying high-permeability zones compared to Arrofi and Abu-Mahfouz [4], who primarily observed surface features. By leveraging the enhanced capabilities of the CET Grid Analysis alongside the FFD method, our analysis revealed five distinct high-density zones, with significant correlation to thermal manifestations such as hot springs. This is particularly evident in Zone A, which not only exhibited the highest FFD but also aligned closely with previously identified geothermal indicators. Such correlations underscore the importance of multi-scale analysis, as demonstrated in our findings, supportive of the conclusions drawn by Rafiq et al. [5] regarding the significance of integrated approaches in geothermal resource assessment.

Furthermore, our approach addresses some limitations noted in earlier works regarding the reliance on surface data alone. For instance, while Arrofi et al. [3] acknowledged the challenges posed by surface noise in lineament detection, our incorporation of geophysical data helps mitigate such issues, allowing for refined identification of subsurface structures that may significantly impact geothermal fluid movement.

### Statistical validation of the results

To quantitatively assess the effectiveness of our protocol in extracting structural lineaments and identifying high-permeability zones within the study area, we have employed the precision metric to evaluate the performance of our lineament extraction methodology on the manually extracted surface structural lineaments. This metric indicates the proportion of true positive identifications (correctly identified lineaments corresponding to high-permeability zones) out of all identified high-permeability lineaments. It reflects the accuracy of our lineament extraction process.

(4) $$\mathrm{Precision}=\frac{TP}{\left(TP+FP\right)}$$

where:

• TP = True Positives (correctly identified lineaments)
• FP = False Positives (incorrectly identified lineaments)

The result of the statistical validation of the manually extracted surface structural lineaments using the precision metric is 87%, indicating that our protocol is of good predictive accuracy.

### Implications

Our protocol is not limited to geothermal exploration, but it is suitable for a range of geological processes, such as mineral exploration, soil stability assessments, hydrogeological studies, and seismic hazards assessment.

Our integrated method of remote sensing and magnetic data analysis can significantly enhance mineral exploration efforts. For instance, the same lineament detection techniques employed in our study can be used to identify fault zones and fractures that are often associated with mineral deposits. Studies like that of Arrofi et al. [3] have demonstrated that certain mineralization processes are closely linked to structural lineaments. By adapting our approach to focus on economic minerals, we can better direct exploratory drilling and resource assessment activities to high-potential areas.

The ability to map subsurface lineaments using magnetic data also has significant implications for evaluating soil stability and landslide risk. Structural lineaments often serve as pathways for groundwater, which can weaken soil cohesion and heighten the likelihood of landslides during heavy rainfall. By employing our protocol to identify high-density lineament zones in hilly or mountainous regions, planners and engineers could better assess potential landslide risks and design appropriate mitigation measures.

Beyond strictly mineral and geothermal contexts, our methodology is applicable in hydrogeology for mapping aquifer systems. Understanding the structural controls on groundwater flow is vital for water resource management. Lineaments with high fracture density may indicate areas of enhanced permeability, crucial for locating groundwater reserves.

Our protocol could also be adapted for seismic hazard assessments. The detection of fault lines and fractures is essential in understanding seismic risk, and our methodology can improve the identification of involved structures within tectonically active regions. By correlating lineament density with historical seismic activity, potential areas of risk can be highlighted for further investigation and monitoring.

### Limitations of the protocol

One of the primary limitations of our protocol is the automation of the lineament detection process. The automatic analysis results should be checked for inaccuracies or structures that have been wrongly interpreted when compared with the geophysical or topographical maps. The validation of the results depends on the parameters used in the different steps of the CET analysis workflow (magnetic anomalies polarity, amplitude threshold, cells where lineaments extent, etc.). Each case study should be treated individually based on a priori geological, tectonic, or geophysical information, as well as the purpose of the study since the use of different parameter values can alter the final results. This limitation for the subsurface lineament analysis is best addressed with the manual interpretation of lineaments using edge-detection techniques, such as the calculation of the tilt derivative (e.g., [7]). However, these methods require more time and are often prone to subjective biases.

Noise present in the data, variance in geological features, and the inherent complexity of the landscape can also lead to errors in the results that need to be dealt with. Noise can lead to false positives or the omission of significant features. Moreover, variations in geological environments, such as differences in vegetation cover, topography, and soil type, can influence the detection algorithms' performance, resulting in inconsistent outcomes.

Another limitation is the dependence on the quality of input data. The effectiveness of our approach is tightly linked to the quality of input data. Poor-quality remote sensing images, inaccuracies in magnetic surveys, or incomplete geological maps can severely limit the robustness of our findings. Issues such as cloud cover in satellite imagery or artifacts in geophysical data can obscure critical information needed for accurate structural analysis.

### Areas for future development

In light of these limitations, we identify several promising avenues for future development:

Exploring advanced machine learning algorithms can significantly enhance our data integration and lineament detection processes. Techniques such as deep learning, which can better discern patterns within large datasets, may improve the accuracy of automated lineament detection by minimizing the effects of noise and enhancing the ability to identify lineaments in complex geological environments.

Conducting field verification studies to assess the accuracy of detected lineaments could provide valuable feedback for refining our methodology. Such studies would help validate the findings and improve the understanding of the relationship between identified features and actual geological structures.

## Conclusions

In conclusion, this study advances the field of geothermal exploration by integrating remote sensing and geophysical methodologies, providing a holistic understanding of structural lineaments and their implications for geothermal resource identification. Our findings illustrate the enhanced capacity for pinpointing high-permeability zones, demonstrating a marked improvement over prior methodologies. By facilitating a more comprehensive analysis of geothermal potential, our protocol not only contributes to the existing literature but also sets the stage for future explorations in structurally complex terrains worldwide.

## Credit author statement

**Jawad Rafiq:** Conceptualization, Methodology, Software, Original draft preparation **Konstantinos Chavanidis**: Validity tests, Data curation, Writing- Software. **Israa S. Abu-Mahfouz**: Visualization, Investigation, Supervision, Validation, Reviewing and Editing, Funding **Pantelis Soupios:** Supervision, Validation, Writing- Reviewing and Editing.

## Funding

This work was supported by the College of Petroleum Engineering and Geosciences (CPG) of the 10.13039/501100004055King Fahd University of Petroleum and Minerals (KFUPM) [Grant number: SF22001].

## Declaration of competing interest

The authors declare that they have no known competing financial interests or personal relationships that could have appeared to influence the work reported in this paper.

## Data Availability

Data will be made available on request.
